# Functional and metabolic impairment in cigarette smoke-exposed macrophages is tied to oxidative stress

**DOI:** 10.1038/s41598-019-46045-7

**Published:** 2019-07-03

**Authors:** Daniel S. Aridgides, Diane L. Mellinger, David A. Armstrong, Haley F. Hazlett, John A. Dessaint, Thomas H. Hampton, Graham T. Atkins, James L. Carroll, Alix Ashare

**Affiliations:** 1 Section of Pulmonary and Critical Care Medicine, Dartmouth-Hitchcock Medical Center, Lebanon, NH USA; 2 Department of Microbiology and Immunology, Geisel School of Medicine, Hanover, NH USA

**Keywords:** Mechanisms of disease, Alveolar macrophages

## Abstract

Cigarette smoke inhalation exposes the respiratory system to thousands of potentially toxic substances and causes chronic obstructive pulmonary disease (COPD). COPD is characterized by cycles of inflammation and infection with a dysregulated immune response contributing to disease progression. While smoking cessation can slow the damage in COPD, lung immunity remains impaired. Alveolar macrophages (AMΦ) are innate immune cells strategically poised at the interface between lungs, respiratory pathogens, and environmental toxins including cigarette smoke. We studied the effects of cigarette smoke on model THP-1 and peripheral blood monocyte derived macrophages, and discovered a marked inhibition of bacterial phagocytosis which was replicated in primary human AMΦ. Cigarette smoke decreased AMΦ cystic fibrosis transmembrane conductance regulator (CFTR) expression, previously shown to be integral to phagocytosis. In contrast to cystic fibrosis macrophages, smoke-exposed THP-1 and AMΦ failed to augment phagocytosis in the presence of CFTR modulators. Cigarette smoke also inhibited THP-1 and AMΦ mitochondrial respiration while inducing glycolysis and reactive oxygen species. These effects were mitigated by the free radical scavenger N-acetylcysteine, which also reverted phagocytosis to baseline levels. Collectively these results implicate metabolic dysfunction as a key factor in the toxicity of cigarette smoke to AMΦ, and illuminate avenues of potential intervention.

## Introduction

Cigarette smoking infuses the respiratory tract with toxins, causing abundant, long-lasting consequences even after smoking cessation^1–3^. Among those consequences is chronic obstructive pulmonary disease (COPD), which is projected to soon be the third leading cause of death worldwide^4,5^. As the principal innate immune cell in the healthy lung, alveolar macrophages (AMΦ) are implicated in dysregulated inflammation and cycles of recurrent and chronic infections of COPD, yet the direct toxicity of cigarette smoke to ΑΜΦ remains incompletely understood^6^. AMΦ are responsible for clearing pathogens, particulate matter, and cellular debris from the lung, and any impairment in this process is likely to have deleterious physiologic consequences^7^. For example, poor phagocytosis is known to negatively correlate with lung function in COPD^8^. Conversely, rescuing diseased AMΦ function may prove of therapeutic benefit; azithromycin has been shown to augment phagocytosis of apoptotic bronchial epithelial cells (also known as efferocytosis) in COPD AMΦ^9,10^. In addition to impaired efferocytosis^9–12^, COPD AMΦ are known to be defective in phagocytosis of bacteria^13–16^, however data on the acute effects of cigarette smoke on AMΦ are lacking.

An emerging area of research into COPD pathophysiology relates to the role of the cystic fibrosis transmembrane conductance regulator (CFTR), a chloride and bicarbonate channel which is mutated in cystic fibrosis (CF)^17^. CFTR dysfunction has been demonstrated in bronchial epithelial cells from COPD patients, presumably contributing to mucous hyperviscosity and ciliary stasis with subsequent mucous plugging, impaction, and chronic infections^18–22^. The CFTR potentiator ivacaftor (also known as VX-770) is FDA approved as monotherapy for CF patients with CFTR gating mutations^23,24^, and is a component of combination therapies for patients with trafficking mutations^25–29^. As such its use has significantly improved outcomes for CF patients^30^. It has also shown promise in reversing cigarette smoke-induced CFTR dysfunction *in vitro* and is currently in clinical trials for patients with chronic bronchitis^31,32^.

CFTR dysfunction has been shown to impair phagocytosis in CF MΦ^33,34^ in a manner that is reversible by the CFTR corrector lumacaftor^35^, raising the possibility that CFTR activity is required for optimal function of COPD MΦ as well. Herein we investigate the effects of cigarette smoke on AMΦ phagocytosis of *Pseudomonas aeruginosa*, CFTR expression, and the potential salutary effects of CFTR modulating drugs.

MΦ phagocytosis is also understood to be intertwined with metabolism and reactive oxygen species^36–39^. Inflammatory activation of MΦ causes them to shunt glucose towards glycolysis in lieu of mitochondrial oxidation, and conversely, modulating metabolism impacts inflammatory state^36,40^. Availability of glucose has been shown to modulate phagocytosis as well as glycolytic rates in an *in vitro* diabetes model^41^. Here we investigate how cigarette smoke impacts MΦ metabolism and reactive oxygen species (ROS) generation, and probe the activity of the free-radical scavenger N-acetylcysteine (NAC) in rescuing smoke-induced dysfunction.

## Results

### Cigarette smoke extract inhibits phagocytosis of *Pseudomonas aeruginosa* by immortalized and primary macrophages

**Figure 1:**
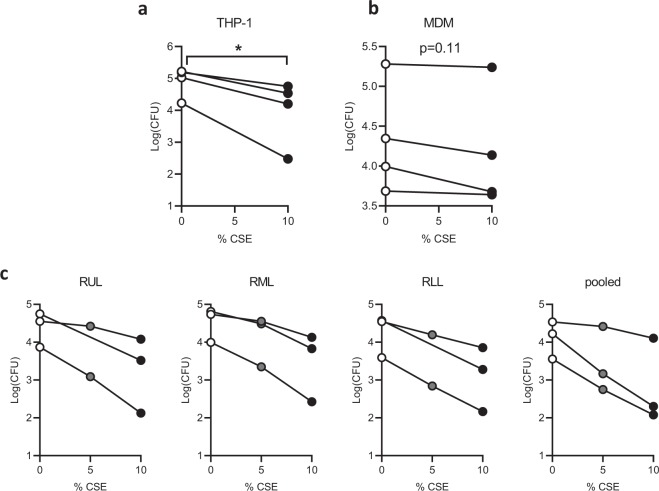
CSE inhibits phagocytosis of *Pseudomonas aeruginosa* by immortalized and primary macrophages. (**a**) THP-1 cells were treated with 50 nM PMA for 48 hours to differentiate into macrophages, then phagocytosis was quantified by a gentamicin protection assay. Cells were cultured in standard medium without antibiotics, pre-treated with 10% CSE or media vehicle for 20 min, then infected with log-phase *P. aeruginosa* at a multiplicity of infection (MOI) of 10 for 20 min. They were then washed and 10x gentamicin was added to kill extracellular bacteria. Three additional washes were performed and cells were lysed in 0.1% triton X-100 in PBS. Colony forming units (CFU) were quantified on LB agar plates and CFU per million macrophages was calculated, log(mean CFU) for four individual experiments are graphed with lines connecting replicates from an individual experiment. *p < 0.05 by paired t-test (**b)** Primary human peripheral blood monocytes were differentiated into monocyte-derived macrophages (MDM) by treatment for 7 days with 100 ng/mL M-CSF, followed by a gentamicin protection assay as in **a**. Means of triplicate technical replicates from four independent experiments (separate donors) are graphed. (**c)** Primary alveolar macrophages (AMΦ) were purified from right upper lobe (RUL), right middle lobe (RML), or RLL (RLL) bronchoalveolar lavage, followed by phagocytosis assays after pretreatment with vehicle, 5% CSE or 10% CSE (5% point was omitted from RUL and RLL replicates of one donor due to limited cell number). Means of triplicate technical replicates from three separate donors are graphed. CSE significantly reduced phagocytosis (p < 10^−15^) based on a linear model of log10 CFU as a function of CSE concentration, experimental batch and location in the lung.

We first determined the effects of cigarette smoke extract (CSE) on phagocytosis of *P. aeruginosa* utilizing a gentamicin protection assay. We chose a 20 min incubation with bacteria, which was previously demonstrated to be within the linear phase of bacterial ingestion^35^. Using THP-1 cells differentiated into MΦ by addition of the protein kinase C agonist phorbol 12-myristate 13-acetate (PMA), we found a large decrease in *P. aeruginosa* uptake after a 20 minute pretreatment with 10% CSE (Fig. 1a), a concentration determined in preliminary experiments to show effects without impacting cell viability as determined by lactate dehydrogenase (LDH) release (Supplementary Fig. S1). In primary human peripheral blood monocyte-derived MΦ (MDM) we saw a smaller trend towards decreased phagocytosis that was not statistically significant (Fig. 1b).

While there are reports describing decreased phagocytosis in AMΦ purified from patients with COPD and “healthy” smokers relative to non-smokers^6,11,13,15^, there are no published reports on the acute effects of CSE on bacterial phagocytosis in healthy human AMΦ. Here we found that CSE induces a dose-dependent decrement in phagocytosis in MΦ from all three right lung lobes as well as pooled MΦ with no significant differences in CSE effect between lobes (Fig. 1c). While there were some differences in the basal rates between experiments on AMΦ from three separate individuals, these likely represented day-to-day experimental variability, as similar variations were seen with immortalized THP-1 cells (Fig. 1a) and MDM (Fig. 1b). Analyzing the AMΦ data with linear models to control for batch effects, we found a highly significant effect of CSE concentration (p < 10^−15^) on phagocytosis. RLL ΑΜΦ had lower baseline phagocytosis levels when compared with RUL (p < 0.05) or RML (p < 0.001) however there was no significant difference in their respective sensitivities to CSE.

### CFTR is diminished by CSE but CFTR modulators fail to impact phagocytosis

**Figure 2:**
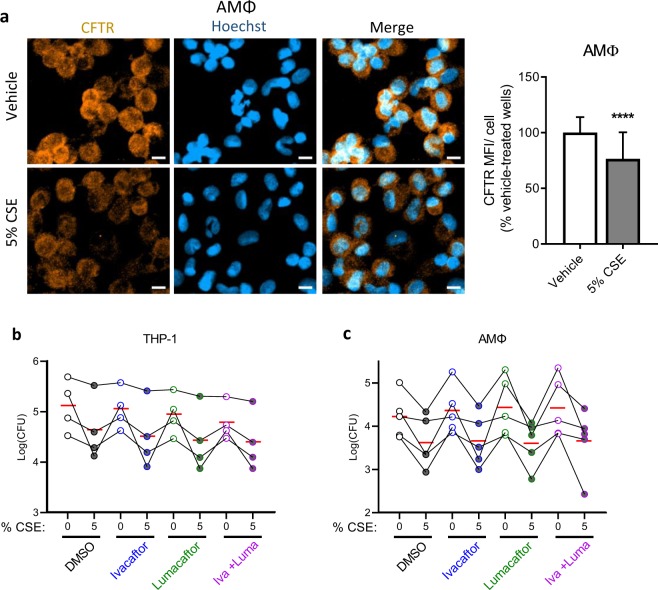
CSE decreases CFTR expression while CFTR modulators fail to rescue phagocytosis. (**a**) Primary AMΦ from healthy donors were plated overnight onto glass slide chambers. The following morning they were treated with vehicle or 5% CSE for one hour. They were then washed, fixed in methanol, and stained for CFTR and counterstained with Hoechst. Three 20x fields in three separate wells for each condition were imaged, mean orange fluorescence was calculated and divided by the number of cell nuclei/field then normalized to vehicle-treated wells. Mean and standard deviations from five independent experiments from different donors with 6–9 fields imaged from each are graphed, ****p < 0.0001 by Mann-Whitney test. Scale bars = 10 μm. (**b**) Phagocytosis assay was performed with THP-1 cells as in Fig. 1 except cells were pre-treated with DMSO, ivacaftor 30 nM, lumacaftor 3 μM or the combination of the two for 48 hrs prior to assay, after differentiation with PMA. Points with connecting lines represent means from four individual experiments, each performed in triplicate. Red horizontal lines indicate mean of the four experiments. Linear models revealed that all three treatments reduced log10 CFU (ivacaftor: p = 0.08; lumacaftor: p < 0.01; 5% CSE: p < 0.001) in THP-1 cells. (**c**) In AMΦ, only 5% CSE was associated with significant reduction in phagocytosis (p < 0.001).

Given the importance of CFTR for phagocytosis^34,35,42^ as well as reported decrements in CFTR expression and function in smoke-exposed epithelial cells^19,21,43–45^, we determined the effects of CSE on CFTR expression in primary AMΦ. We exposed AMΦ to 5% CSE as this induced an intermediate inhibition of phagocytosis and was therefore felt to be an appropriate concentration for downstream experiments. We reasoned that if CFTR was relevant to phagocytosis under the short-term conditions in Fig. 1, its expression should be affected in a similar timeframe. We therefore chose a one hour timepoint based upon the total time from the start of CSE exposure in the phagocytosis assays (20 minute CSE preincubation, 20 minute infection, and 15 minute antibiotic treatment). Under these conditions we find that 5% CSE significantly reduced total CFTR signal per cell as measured by immunofluorescence (Fig. 2a).

As lumacaftor was recently shown to rescue phagocytosis efficiency in monocyte-derived macrophages from CF donors^35^, we sought to determine the effects of CFTR modulators on CSE-induced defects. Using previously published concentrations of ivacaftor (30 nM) and lumacaftor (3 μM) which were in turn derived from serum levels found in CF subjects^35^, we pre-treated THP-1 derived MΦ (Fig. 2b) or primary human AMΦ (Fig. 2c) with ivacaftor, lumacaftor, or their combination (as in the FDA approved CF drug formulation Orkambi®) for 48 hours. This extended pre-incubation was to allow for the slower effects of the CFTR corrector lumacaftor. Under these conditions we found that lumacaftor significantly reduces THP-1 phagocytosis (p < 0.01), and a marginally significant trend (p = 0.099) suggesting that ivacaftor may also reduce phagocytosis. As expected, there was a highly significant (p < 0.001) reduction in phagocytosis by 5% CSE. We confirmed that over a wide range of concentrations from 10 nM through 10 μM there was no dose-dependent effect of either compound on THP-1 phagocytosis in the presence or absence of CSE (Fig. S2). In the presence of CSE, CFTR modulators showed no effect on AMΦ phagocytosis in any of individuals tested (Fig. 2c).

### CSE shifts MΦ metabolism from oxidative phosphorylation to glycolysis

**Figure 3:**
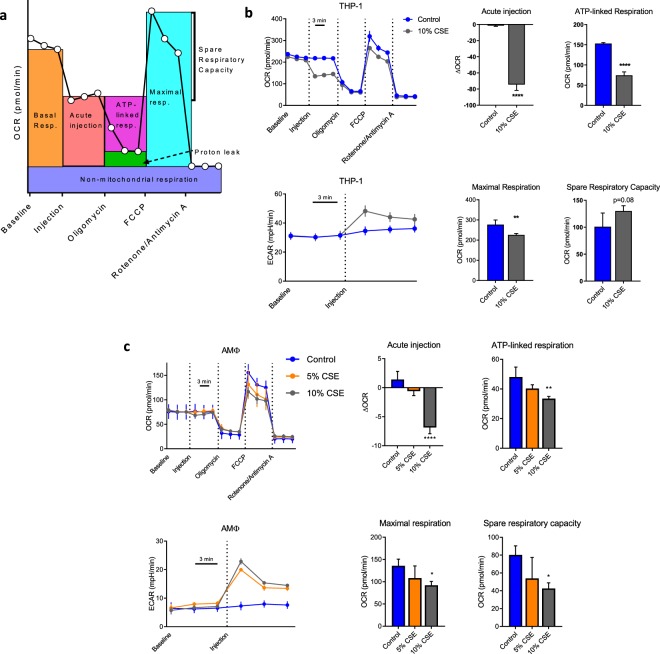
CSE induces a shift in MΦ from oxidative to glycolytic metabolism. (**a**) Schematic of parameters measured in mitostress assay, adapted from^63^ (**b**) THP-1 derived MΦ were subjected to a Seahorse mitostress assay per manufacturer protocol. Either CSE or vehicle was added to injection port A of the Seahorse plate, with a final concentration of 10% after injection. Oligomycin (final concentration 1 μM), FCCP (0.5 μM) and Rotenone/antimycin A (0.5 μM each) were added to injection ports B, C and D. Oxygen consumption rate (OCR, top panel) and extracellular acidification rate (ECAR, right panel) were measured simultaneously three times each at baseline and after each serial injection with three minute intervals between measurements. 3–4 technical replicates per condition were run and mean ± s.d. are graphed, data are representative of three independent experiments. Bar graphs are data computed from OCR line graph with parameters as per **a**. All calculations were performed relative to values from a given well. **p < 0.01, ****p < 0.0001 by unpaired t-test. (**c**) Alveolar macrophages purified from a healthy volunteer and subjected to an identical mitostress assay as in (**b**), excepting that injection was with 5% or 10% CSE. Six technical replicates per condition were run and mean ± s.d. are graphed, data are representative of three unique experiments performed with cells from different donors. *p < 0.05, **p < 0.01, ****p < 0.0001 relative to control wells by ANOVA with Dunnett’s post-test.

Given the lack of effect of ivacaftor and lumacaftor on the phagocytosis defect, we sought to understand what other pathways besides CFTR dysfunction were involved. MΦ metabolism has been demonstrated to be integral to function^36,46,47^, therefore we measured oxygen consumption and proton production (as proxies for oxidative phosphorylation and glycolysis, respectively) with an extracellular flux analyzer after CSE injection. We performed a mitochondrial stress assay which allows for the quantitation of basal respiration, ATP-linked respiration, maximal respiration, proton leak and non-mitochondrial respiration^48^. Figure 3a presents a schematic of the parameters measured with the assay. As seen in Fig. 3b, addition of CSE to THP-1 MΦ immediately inhibited ATP-linked respiration with a compensatory increase in glycolysis. There were no differences in the proton leak or non-mitochondrial respiration, however there was a small decrease in the maximal respiratory rate. Primary AMΦ meanwhile, exhibited a smaller decrement in ATP-linked respiration with CSE, with similar to increased relative glycolysis induction (Fig. 3c). AMΦ demonstrated a comparable decrease in maximal respiration to THP-1 cells. The discordant effects on spare respiratory capacity between the cell types are attributable to the stronger inhibition of respiration in THP-1 cells (acute injection) relative to maximal respiratory capacity. Pre-treatment of either THP-1 or AMΦ with ivacaftor, lumacaftor or their combination had no effect on any of the parameters studied here (Supplementary Fig. S3).

### Cigarette smoke induces reactive oxygen species

**Figure 4:**
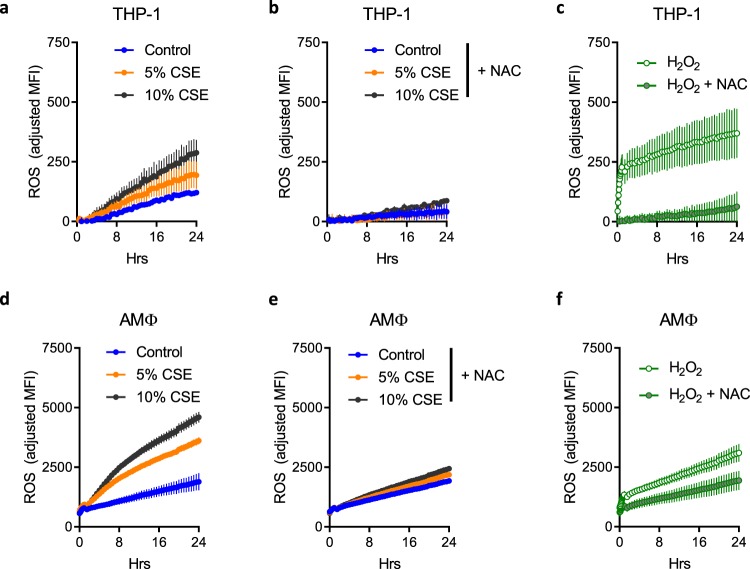
CSE induction of ROS is mitigated by NAC. THP-1 derived MΦ were plated in 96-well plates, then loaded with 10 μM DCF-DA for 30 min. Cells were washed once with PBS and treated with vehicle (**a**) or NAC 4 mM (**b,c**) as indicated for 30 min, followed by the addition of vehicle, CSE or H_2_O_2_ (250 μM). Fluorescence readings with excitation at 488 nm and emission 523 nm were taken every 5 min for one hour then every 30 min for 23 hrs. Readings were normalized to those of identically treated wells without fluorescent dye. Plots from (**a**–**c)** are derived from a single experiment, data were separated onto three graphs for clarity. Four wells per condition were run and mean ± s.d. graphed, data are representative of three independent experiments. **(d**–**f)** Experiments were performed as above using primary AMΦ from a healthy donor. Data are representative of unique experiments on cells from three separate donors with six technical replicates each.

As mitochondrial dysfunction can result in increased production of ROS due to incomplete reduction of oxygen^49^, we tested the presence of free radicals using the probe 2′,7′-dichlorofluorescin diacetate (DCF-DA), which is a ROS-activated fluorophore. There was a dose-dependent increase in the production of ROS by THP-1 MΦ in the presence of CSE (Fig. 4a) which was inhibited by the free radical scavenger NAC (Fig. 4b) and reproduced by the exogenous addition of hydrogen peroxide as a positive control (Fig. 4c). Figure 4a–f each represent data from one experiment which was graphed onto three separate plots for clarity due to overlap of the data. When cultured in reducing medium, primary AMΦ exhibited a similar pattern of ROS production to THP-1 MΦ however with approximately an order of magnitude greater signal (Fig. 4d–f).

### N-acetylcysteine improves metabolic dysfunction in primary AMΦ

**Figure 5:**
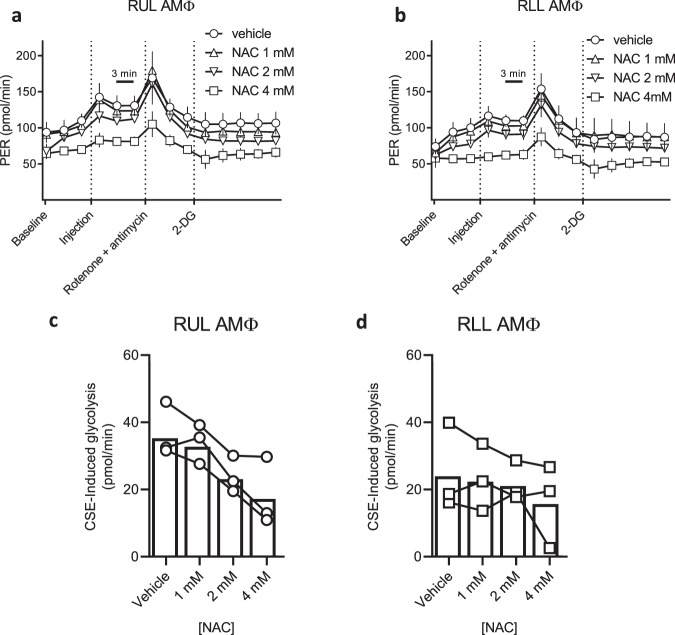
NAC blunts the CSE-induced metabolic shift in primary upper and lower lobe AMΦ. (**a**) RUL and (**b**) RLL AMΦ were purified from a healthy volunteer and plated overnight. Wells were pre-treated with vehicle or NAC at the indicated concentrations for 30 min prior to standard glycolytic rate assay performed according to manufacturer instructions. CSE was placed in injection port A of the Seahorse assay (final concentration 5% after dilution). Glycolytic rate assay was then run and proton efflux rate (PER) quantified at three minute intervals. 5–6 technical replicates per condition were run and mean ± s.d. graphed. Data are representative of three unique experiments with cells from separate donors. (**c**,**d**) CSE-induced glycolysis was calculated in RUL and RLL AMΦ, respectively, from three independent donors. Symbols represent means of 5–6 technical replicates per experiment, and bars indicate combined means of the three donors. CSE-induced glycolysis is reduced in a dose dependent fashion by NAC concentration in both the RUL (p < 0.0001) and RLL (p < 0.05) based on linear models.

Mitochondrial dysfunction and ROS can form a feedback loop whereby mitochondrial ROS can further inhibit mitochondrial function^50^, therefore we tested the ability of NAC to interrupt this cycle by pre-treating AMΦ prior to CSE injection. Preliminary experiments with the mitochondrial stress assay demonstrated significant artefact in the presence of NAC, potentially due to direct buffering effects in the otherwise unbuffered media; we therefore chose to use a glycolytic rate assay (buffered with 5 mM HEPES) for further investigation. Figures 5c,d illustrate how NAC decreases CSE-induced glycolysis in a dose-dependent manner. AMΦ from upper and lower lobes are known to exhibit differential inflammatory responses to hypoxia^51^ which is a known inducer of oxidative stress, however we saw similar effects in right upper lobe and right lower lobe AΜΦ.

### CSE phagocytosis inhibition is replicated by hydrogen peroxide and reversed by NAC

**Figure 6:**
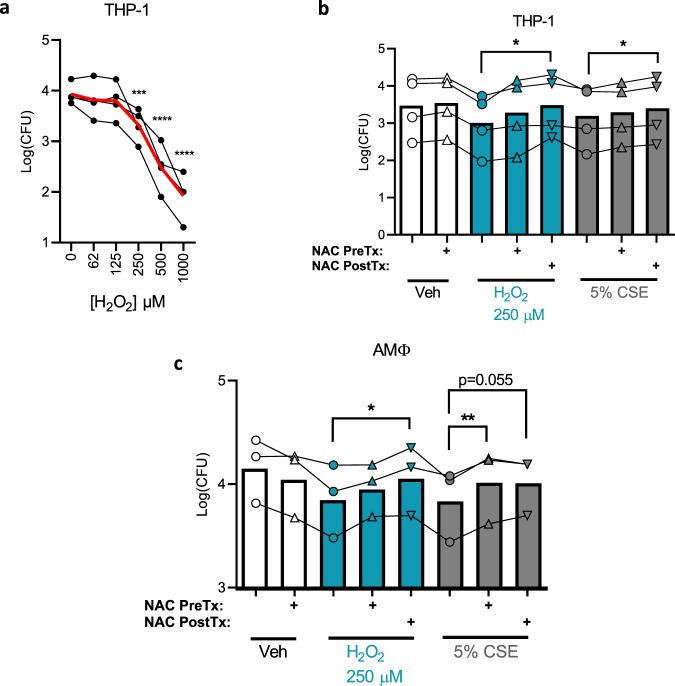
Reactive oxygen species inhibit phagocytosis. (**a**) Phagocytosis was carried out as in Fig. 1 except varying doses of hydrogen peroxide were used to pre-treat cells for 30 min prior to infection. Phagocytosis generally decreases with increasing hydrogen peroxide concentration (p < 10^−9^, linear model). Means of four independent experiments are graphed (black dots) with the mean of all four experiments combined (red line). ***p < 0.001, ****p < 0.0001 compared with control by ANOVA with Dunnett’s post-hoc test. p < 10^−9^ for an effect of hydrogen peroxide concentration on phagocytosis as determined by linear modeling. (**b**) Phagocytosis assay similar to **a** except NAC 4 mM was added to indicated wells either 30 min before hydrogen peroxide/CSE (NAC PreTx) or 30 min after (NAC PostTx). Infection was started 30 min after post-treatment. Points represent means of four independent experiments each performed in triplicate, with bars indicating the overall means of the combined experiments. *p < 0.05 by paired t-test. (**c**) Identical phagocytosis assays to (**b**) were carried out using AMΦ from healthy donors, points represent means of three unique experiments from distinct donors each performed in triplicate, with bars indicating the overall means of the combined experiments. *p < 0.05, **p < 0.01 by paired t-test.

The data presented thus far suggest a connection between the metabolic and functional defects induced by CSE in THP-1 and alveolar MΦ, therefore we tested the hypothesis that CSE-induced ROS are directly interfering with phagocytosis. Hydrogen peroxide replicated the inhibitory effect of CSE on phagocytosis in THP-1 cells, causing a dose-responsive decrement in the phagocytosis efficiency (Fig. 6a), with a comparable effect to 5% CSE at 250 μM. Linear models revealed a highly significant (p < 10^−9^) dose-dependent effect of hydrogen peroxide. Further implicating ROS in this process, NAC reversed the phagocytosis defect of either hydrogen peroxide or CSE (Fig. 6b). In THP-1, NAC had a greater effect when it was added to the medium after the hydrogen peroxide or CSE (NAC PostTx) rather than before (NAC preTx), reaching statistical significance in both cases by paired t-tests, whereas pre-treatment with NAC only produced a trend towards significance. Linear models demonstrate that both hydrogen peroxide (p < 0.0001) and 5% CSE (p < 0.01) decrease phagocytosis, whereas NAC post-treatment has a positive effect (p < 0.0001). Experiments using primary AMΦ from three donors revealed broadly similar results with subtle differences. In this model, NAC post-treatment continued to reverse the effects of hydrogen peroxide, however in CSE-treated cells, NAC pre-treatment reached statistical significance whereas NAC post-treatment fell just short of this threshold (p = 0.055).

## Discussion

While there is little doubt that AMΦ dysfunction plays a key role in COPD, there is less known about the mechanisms thereof, making it challenging to target therapeutically^8^. We originally hypothesized that CFTR may play a role, as it has been previously shown to be important for CF ΜΦ^35^. In epithelial cells CFTR is known to be inhibited by cigarette smoke, both at the channel function and protein expression levels^43,44,52^. It has been previously reported in RAW 264.7 murine MΦ that *P. aeruginosa* phagocytosis is blocked by CSE in a partially CFTR-dependent manner^53^, however there were methodological differences in their CSE preparation and phagocytosis assays including MOI and length of infection, in addition to the cell type studied. In the presence of CSE, these authors saw increased clearance of bacteria from cell culture media with the preclinical CFTR potentiator and corrector VRT-532, which occurred without an appreciable increase in CFTR protein levels. Unfortunately, two clinically available CFTR modulators in our hands were ineffective at improving phagocytosis. The same group showed that oxidative stress is connected to impaired CFTR trafficking in smoke-exposed epithelial-like cells, leading to aggresome formation and degradation of misfolded proteins^54^. Whether or not similar CFTR trafficking pathways are involved in smoke-exposed MΦ remains to be seen, as well as whether they are amenable to targeting by CFTR modulators. It is important to note that our data do not demonstrate that CFTR is irrelevant to phagocytosis and/or metabolism in smoke-exposed macrophages, only that two CFTR modulators were unable to reverse the acute insult of CSE exposure on macrophage function. Complementary approaches using CFTR mutants and knockouts are currently underway in our laboratory to better address this question. A second CFTR corrector, tezacaftor, has recently been approved, and other CFTR modulators with different mechanisms are in the pipeline which will increase the pharmacologic arsenal and could have different results^25,26^.

Regarding the relationship between CFTR and phagocytosis, we postulate that the CFTR decrement seen here is a separate downstream effect of CSE, and therefore targeting more proximal events such as oxidative stress is likely to be of higher yield than targeting CFTR itself. Indeed NAC proved more effective at reversing the phagocytosis defect in our system, as well as metabolic consequences of CSE. NAC was even able to improve phagocytosis when added after smoke or hydrogen peroxide exposure (Fig. 6b,c), increasing its therapeutic potential.

NAC has had mixed results as a treatment for COPD; while there are some data that it can decrease exacerbations at high doses in a cohort of patients who are subject to frequent exacerbations^55,56^, the effects are modest at best, while other studies have shown no benefit^57^. This may be due to the high (mM) concentrations needed to produce effects *in vitro*, which could be difficult to attain *in vivo*.

While the experimental *ex vivo* system presented herein has strengths in that it allows us to evaluate the acute effects of CSE, it is also limited by that acuity. Administration of CSE is quite different from the chronic exposure to which smokers’ lungs are subject, and it will be interesting to see the relative contributions of CFTR and oxidative stress in smoker and COPD AMΦ. These two pathways are not mutually exclusive as CFTR itself is known to be a glutathione transporter^58,59^, and oxidative stress is exacerbated by CFTR dysfunction impairing the normal glutathione response^60^. Oxidative stress can then further decrease CFTR levels^61^, forming a vicious cycle. Experiments to illuminate these possibilities are ongoing in our laboratory. The source and identity of the elevated ROS will likewise be informative.

In summary, we find that cigarette smoke acutely inhibits bacterial phagocytosis as well as mitochondrial function in human AMΦ. While CSE also decreases CFTR levels, CFTR modulators ivacaftor and lumacaftor are unable to rescue CSE defects. Meanwhile, NAC partially reverses the dysfunction, consistent with increased oxidative stress seen in the presence of CSE. This provides further basic mechanistic rationale for the use of antioxidants in COPD, somewhat tempered by the high concentrations needed to see an effect *in vitro*. Further studies will extend this work into primary AMΦ isolated from current smokers.

## Methods

### Human subjects

This study was approved by the Committee for the Protection of Human Subjects at the Geisel School of Medicine at Dartmouth (CPHS protocol #22781), and all procedures were performed in accordance with relevant guidelines and regulations. Healthy subjects (n = 24 total, 3–5 per experiment) were enrolled if they were between 18 and 55 years of age, non-smokers, and had no underlying pulmonary disorder or significant comorbidity. Informed consent was obtained from all participants for participation in research, phlebotomy, and for bronchoscopy individually. Phlebotomy (100 mL whole blood for monocyte isolation) was performed at the time of peripheral IV insertion. Next, after local anesthesia to the posterior oropharynx, with or without systemic sedation per patient preference, a flexible bronchoscope was passed through the mouth and vocal cords until wedged into sub-segmental bronchi of the right lung. Five lavages each of 20 cc 0.9% saline and 10 cc air were performed in the right upper lobe, right lower lobe, and right middle lobe sequentially. Bronchoalveolar lavage (BAL) samples from individual lobes were obtained and alveolar macrophages purified as previously described^51^.

## Materials

Sources of reagents are available in the online Supplementary Information.

### Generation of cigarette smoke extract

CSE was purified according to protocol of Blue and Janoff as described by others^62^. One cigarette was lit and attached to 5 cm plastic tubing and placed on the end of a 60 mL syringe containing 10 mL of media. Smoke was drawn into the chamber of the syringe until 50 mL total volume was obtained, then agitated until the smoke cleared. This process was repeated until the cigarette was consumed, which was defined as 100% CSE.

### Phagocytosis assay

A modified gentamicin protection assay^35^ was used to quantify phagocytosis. Specific details are available in the Supplementary Information.

### Immunofluorescence

Primary alveolar macrophages were plated overnight, then treated with 5% CSE for one hour and stained for CFTR, followed by counterstaining with Hoechst to reveal nuclei. Specific details are available in the Supplementary Information.

### Extracellular flux assay

Macrophages were plated onto Seahorse assay plates obtained from the manufacturer at 50,000/well. Assay was run according to manufacturer’s protocol for mitostress assay and glycolytic rate assay, respectively. CSE was added to port A to be injected prior to kit compounds in ports B through D respectively. Reagent concentrations and buffer composition are in the Supplementary Information.

### Reactive oxygen species assay

Macrophages were loaded with 10 μM 2′,7′-dichlorofluorescin diacetate (DCF-DA) for 30 min, washed once with PBS and treated with vehicle or NAC 4 mM for 30 min, followed by the addition of CSE or H_2_O_2_ (250 μM). AMΦ were cultured in the presence of 50 μM β-mercaptoethanol to reduce basal ROS. Cells were incubated in a Tecan plate reader at 37 °C and fluorescence readings (excitation 488 nm, emission 523 nm) were taken every 5 min for one hour then every 30 min for 23 hrs. Readings of identically treated wells without dye were subtracted to normalize for fluorescence from media.

### Statistics

All graphs were prepared with GraphPad Prism 7.0 (San Diego, CA). Linear models were run in R as indicated in text and figure legends. ANOVA and Mann-Whitney tests were performed in Prism. All p values are two-tailed and p < 0.05 was interpreted to be statistically significant. All data generated or analyzed during this study are included in this published article (and its Supplementary Information files).

## Supplementary information

Online supplement
