# Supplementary material for: Functional and metabolic impairment in cigarette smoke-exposed macrophages is tied to oxidative stress
Source: Sci Rep. 2019 Jul 3;9:9624. doi: 10.1038/s41598-019-46045-7 (PMC6610132; doi:10.1038/s41598-019-46045-7)
Supplement: Supplementary file 1 — Online supplement [file 41598_2019_46045_MOESM1_ESM.docx]

**Functional and metabolic impairment in cigarette smoke-exposed macrophages is tied to oxidative stress**

**Daniel S. Aridgides^1^, Diane L. Mellinger^1^, David A. Armstrong^1^, Haley F. Hazlett^2^, John A. Dessaint^1^, Thomas H. Hampton^2^, Graham T. Atkins^1^, James L. Carroll^1^, and Alix Ashare^1,2*^**

**^1^Section of Pulmonary and Critical Care Medicine, Dartmouth-Hitchcock Medical Center, Lebanon, NH**

**^2^Department of Microbiology and Immunology, Geisel School of Medicine, Hanover, NH**

*** Correspondence:** [Alix.Ashare@hitchcock.org](mailto:Alix.Ashare@hitchcock.org)

**Supplementary Information**

**Materials**

Phorbol 12-myristate 13-acetate (PMA), 2′,7′-dichlorofluorescin diacetate (DCF-DA), N-acetylcysteine (NAC), Triton X-100, LB, LB agar, dimethylsulfoxide (DMSO), and hydrogen peroxide were from Sigma-Aldrich (St. Louis, MO). Phosphate buffered saline (PBS) and tissue culture plates were from Corning (Corning, NY). Ivacaftor and lumacaftor were from Selleckchem (Houston, TX). Gentamicin and fetal bovine serum were from Genesee Scientific (El Cajon, CA). RPMI + Glutamax, Nunc glass slides and coverslips, prolong gold, Alexa-555 conjugated secondary antibody (catalog number A21422), Hoechst stain, and LDH assay were from ThermoFisher (Waltham, MA). Anti-CFTR Ab (catalog no. sc-376683) was from Santa Cruz (Dallas, TX). Fetal bovine serum (FBS) was from Genesee Scientific (San Diego, CA). Pan-monocyte isolation kit and M-CSF were from Miltenyi biotech (Bergisch Gladbach, Germany). Primocin was from Invivogen (San Diego, CA). THP-1 cells were obtained from ATCC (Manassas, VA). *Pseudomonas* PA14 was a gift from the lab of Deborah Hogan (Dartmouth Geisel School of Medicine). L&M filtered cigarettes (Phillip Morris, Richmond VA) were purchased locally. Mitostress and glycolytic rate assay kits were from Agilent (Santa Clara, CA).

**Cell culture**

THP-1 cells were maintained in RPMI with 10% FBS, 50 μM β-mercaptoethanol, and gentamicin 50 μg/mL. THP-1s are maintained at a density of 2-8 x 10^5^ cells/mL in 75 cm^2^ flasks, and viability is monitored and is routinely >95%. They are used for 2-3 months before a new vial is thawed from liquid nitrogen-frozen aliquots. THP-1s were differentiated into MΦ by treatment with 50 nM PMA for 48 hrs. This was based upon the protocol of Lund et al. without the 24 hour washout period^1^. Peripheral blood monocytes were isolated from whole blood of healthy volunteers as previously described^2^, and differentiated into MΦ by treatment with M-CSF 100 ng/mL in RPMI/10% FBS/gentamicin for 7 days prior to use. AMΦ were plated overnight after bronchoscopy and purification in RPMI with glutamax and primocin 100 μg/mL prior to use.

**Phagocytosis assay**

*Pseudomonas aeruginosa* PA14 strain was cultured overnight in LB broth prior to the assay at 37^o^ C with agitation in a tube angled at 30 degrees. A 1:10 subculture was performed 1 hour prior to phagocytosis assay to obtain log-phase bacteria. At that time, bacteria were pelleted in a tabletop microcentrifuge, washed twice with antibiotic-free cell culture medium, and quantified by optical densitometry at 600 nm (OD600 of 1.0 = 2x10^9^ bacteria/mL). Meanwhile, macrophages were plated at 2.5 x 10^5^/mL then medium was replaced with identical antibiotic-free medium the morning of the assay. Where indicated, DMSO, ivacaftor (30 nM), and/or lumacaftor (3 μM) were added for 48 hrs prior to assay, and replaced whenever medium was exchanged. CSE was added to the wells without exchanging medium for 20 min prior to infection. H_2_O_2_ was substituted for CSE where indicated. For N-acetylcysteine experiments, NAC was added 30 min before (preTx) or 30 min after (postTx) CSE or H_2_Ο_2_.

*Pseudomonas* was then added at a multiplicity of infection (MOI) of 10 for 20 min to allow internalization, washed with PBS once then incubated with cell culture medium with 10x gentamicin added for 15 min to kill extracellular bacteria, then washed twice in PBS for 2 min each at 4^o^ C with gentle shaking. Cells were then lysed with PBS/0.1% Triton X-100 for 15 min at 4^o^ C (500 μL per well on a 12-well plate). 10 μL of lysate was then streaked on an LB agar plate and incubated overnight at 37^o^ C. Colony forming units were counted, phagocytosis rates per one million macrophages were computed, and CFU were then log transformed for graphing.

**Immunofluorescence**

Primary AMΦ were plated overnight, then treated with 5% cigarette smoke extract for one hour. They were then washed with PBS, fixed in methanol for 5 min at -20^o^ C, washed, blocked for one hour at room temperature with PBS/10% goat serum, washed, and incubated overnight at 4^o^ C with anti-CFTR (1:100 in 1.5% goat serum). The following morning they were washed thrice with PBS and incubated with goat anti-mouse-Alexa555 (1:500 in 1.5% goat serum) for one hour at RT. They were washed three more times, stained with Hoechst (10 μg/mL, 5 min), washed again and mounted in Prolong Gold and allowed to cure for 7 days prior to imaging. Images were obtained with a Zeiss confocal microscope using Zen software and a 20x objective. Ten 0.82 μm thick cuts were obtained per field, and images flattened by averaging signal intensity through the z-stack. Mean fluorescence was quantified in each field and divided by the number of nuclei. Three fields per well were obtained times three wells per condition per experiment for quantitation. All imaging parameters were identical between conditions.

**Extracellular flux assay reagents**

CSE final concentration was 5 or 10%, Oligomycin was 1 μM, carbonyl cyanide-p-trifluoromethoxyphenylhydrazone (FCCP) was 0.5 μM, Rotenone/Antimycin A were 0.5 μM each, and 2-decoxyglucose was 50 mM (for glycolytic rate assay). Depending on the number of cells available, 4-8 wells per condition were run per experiment. Assay was run in an Agilent Seahorse XFe96 analyzer. For mitostress assays XF base medium without phenol red was used, for glycolytic rate assay 5 mM HEPES was added.

**Cytotoxicity assay**

THP-1 derived macrophages were treated with CSE on a 12-well plate. After 20 min, 2 hrs, and 24 hrs an aliquot of supernatant was collected, spun down to remove cells and debris, transferred to a new tube, and frozen at -20^o^ C. We chose these timepoints to represent the short (20 min) and medium-term (2 hour) exposures to which our cells would be subjected in our functional assays, and added a 24-hour timepoint to look for more subacute effects. At the 24 hr timepoint, monolayers were treated with lysis buffer per manufacturer instructions to quantify maximal LDH in cultured cells. Cell lysates were likewise cleared by centrifugation and frozen. The following day, an LDH colorimetric assay was performed according to manufacturer instructions. Values obtained using media alone were subtracted from each point. For AMΦ experiment, supernatants from one phagocytosis assay were tested after being cleared by centrifugation and frozen at -20^o^ C.

**Supplemental References**

1. Lund, M. E., To, J., O’Brien, B. A. & Donnelly, S. The choice of phorbol 12-myristate 13-acetate differentiation protocol influences the response of THP-1 macrophages to a pro-inflammatory stimulus. *J. Immunol. Methods* **430**, 64–70 (2016).

2. Barnaby, R. *et al.* Lumacaftor (VX-809) restores the ability of CF macrophages to phagocytose and kill Pseudomonas aeruginosa. *Am. J. Physiol. Lung Cell. Mol. Physiol.* ajplung004612017 (2017). doi:10.1152/ajplung.00461.2017

**a b**

**Supplementary Figure S1. CSE is not cytotoxic to macrophages at low doses**

**a)** THP-1 derived macrophages were treated with increasing doses of CSE on a 12-well plate. LDH measurements at 20 min, 2 hr and 24 hr were performed, and cells were lysed at 24 hr to determine maximal LDH release. Points were performed in triplicate and mean +/- s.d. are graphed. **b)** Supernatants from phagocytosis assay in Fig. 1d were tested for LDH release after 20 min CSE followed by 20 min *Pseudomonas* infection. LDH positive control is shown on the right, points were performed in triplicate and mean + s.d. graphed. Differences between experimental groups are not significant by ANOVA with Tukey’s post-hoc test.

**b**

**a**

**Figure S2. Ivacaftor and Lumacaftor do not affect THP-1 phagocytosis over a wide range of concentrations with or without CSE exposure**

THP-1 macrophages were treated with varied concentrations of Ivacaftor (**a**) or Lumacaftor (**b**) for 48 hours. They were then subjected to a phagocytosis assay as in Fig. 1a, Ivacaftor or Lumacaftor was replaced after media exchange and was present throughout the phagocytosis experiment. Four technical replicates per condition were performed and mean and SEM of three independent experiments are graphed. No concentrations of either drug resulted in a statistically significant deviation from control DMSO treated conditions either in the presence or absence of 5% CSE by two way ANOVA.

**a**

**b**

**Supplementary Figure S3. CFTR modulators have no effect on CSE-induced metabolic shifts**

**a)** THP-1 derived MΦ or **b)** primary AMΦ were cultured for 48 hrs in the presence of ivacaftor 30 nM, lumcaftor 3 μM, both or DMSO prior to standard mitostress assay as in **Fig. 3** with injection of CSE (10% CSE for THP-1 and 5% for AMΦ). Data are representative of three independent experiments, each performed with 4-8 wells per condition.
